# Macroscale structural changes of thylakoid architecture during high light acclimation in *Chlamydomonas reinhardtii*

**DOI:** 10.1007/s11120-023-01067-1

**Published:** 2024-01-05

**Authors:** Mimi Broderson, Krishna K. Niyogi, Masakazu Iwai

**Affiliations:** 1 Department of Plant and Microbial Biology, University of California, Berkeley, CA 94720 USA; 2 Howard Hughes Medical Institute, University of California, Berkeley, CA 94720 USA; 3 Molecular Biophysics and Integrated Bioimaging Division, Lawrence Berkeley National Laboratory, Berkeley, CA 94720 USA

**Keywords:** Airyscan microscopy, Live-cell imaging, Photosynthesis, Thylakoid structure, Photoacclimation, *Chlamydomonas reinhardtii*

## Abstract

Photoprotection mechanisms are ubiquitous among photosynthetic organisms. The photoprotection capacity of the green alga *Chlamydomonas reinhardtii* is correlated with protein levels of stress-related light-harvesting complex (LHCSR) proteins, which are strongly induced by high light (HL). However, the dynamic response of overall thylakoid structure during acclimation to growth in HL has not been fully understood. Here, we combined live-cell super-resolution microscopy and analytical membrane subfractionation to investigate macroscale structural changes of thylakoid membranes during HL acclimation in *Chlamydomonas*. Subdiffraction-resolution live-cell imaging revealed that the overall thylakoid structures became thinned and shrunken during HL acclimation. The stromal space around the pyrenoid also became enlarged. Analytical density-dependent membrane fractionation indicated that the structural changes were partly a consequence of membrane unstacking. The analysis of both an LHCSR loss-of-function mutant*, npq4 lhcsr1*, and a regulatory mutant that over-expresses LHCSR, *spa1-1*, showed that structural changes occurred independently of LHCSR protein levels, demonstrating that LHCSR was neither necessary nor sufficient to induce the thylakoid structural changes associated with HL acclimation. In contrast, *stt7-9*, a mutant lacking a kinase of major light-harvesting antenna proteins, had a slower thylakoid structural response to HL relative to all other lines tested but still showed membrane unstacking. These results indicate that neither LHCSR- nor antenna-phosphorylation-dependent HL acclimation are required for the observed macroscale structural changes of thylakoid membranes in HL conditions.

**Supplementary Information:**

The online version contains supplementary material available at 10.1007/s11120-023-01067-1.

## Introduction

Understanding natural photosynthetic systems is crucial for developing bioengineering technologies for bioenergy research. In green algae and plants, photosynthetic electron transport is initiated by the absorption of light energy mainly by light-harvesting complex (LHC) proteins in chloroplast thylakoid membranes (Jansson 1999; Wobbe et al. 2016). Light energy is essential for photosynthesis, but excess amounts of absorbed energy can cause photooxidative damage to photosystem II (PSII), which lowers photosynthetic efficiency and growth (Aro et al. 1993; Li et al. 2009; Pinnola and Bassi 2018). Photosynthetic organisms have evolved photoprotective mechanisms, collectively called nonphotochemical quenching (NPQ), which harmlessly dissipates excess excitation energy as heat and mitigates photodamage (Muller et al. 2001; Ruban 2016). There are at least five types of NPQ processes according to the time scales of the induction and relaxation kinetics, namely qE, qZ, qT, qH, and qI. The fast-acting component of NPQ on a time scale of seconds to minutes is qE, which is tightly regulated by a proton gradient across the thylakoid membrane (∆pH). On a time scale of minutes, qZ is activated through the conversion of violaxanthin into zeaxanthin bound to LHC antenna proteins (Niyogi et al. 1997a, 1997b). On a similar time scale, qT occurs through the phosphorylation of LHC antenna proteins of PSII (LHCII) (Rochaix 2011; Goldschmidt-Clermont and Bassi 2015). The slowly reversible components of NPQ are qH and qI, which occur in LHCII (Bru et al. 2022) and the PSII reaction center (Nawrocki et al. 2021), respectively.

In the model unicellular green alga *Chlamydomonas reinhardtii*, the stress-related LHC proteins, called LHCSRs (LHCSR1 and LHCSR3), are essential for the qE component of NPQ (Peers et al. 2009). The lack of *LHCSR3* genes (*LHCSR3.1* and *LHCSR3.2*) eliminates most qE (Peers et al. 2009), whereas the lack of *LHCSR1* gene does not cause a severe loss of qE (Peers et al. 2009; Truong 2011). The lack of both LHCSR1 and LHCSR3 causes the complete loss of qE (Truong 2011). Similar to the majority of LHC antenna proteins, LHCSRs are membrane proteins with three transmembrane helices and contain chlorophylls (Chls) and carotenoids (Bonente et al. 2011; Liguori et al. 2013). The qE facilitated by LHCSRs is regulated by the protonation state of acidic residues on the lumenal side of the protein, and this protonation state responds to changes in ∆pH under high light (HL) conditions (Ballottari et al. 2016; Dinc et al. 2016; Troiano et al. 2021). The expression of *LHCSR* genes and the capacity for qE in *Chlamydomonas* are very limited in low light (LL) (Peers et al. 2009; Petroutsos et al. 2011; Maruyama et al. 2014), but they are upregulated by not only HL but also UV-B and blue light, even at low intensities (Allorent et al. 2016; Petroutsos et al. 2016; Redekop et al. 2022). *LHCSR3* gene expression is also correlated with the intracellular CO_2_/inorganic carbon level and is upregulated under low CO_2_ levels (Redekop et al. 2022; Águila Ruiz-Sola et al. 2023).

For the induction of qZ, the violaxanthin de-epoxidase (VDE) needs to be activated by a high ∆pH across the thylakoid membrane and convert violaxanthin to zeaxanthin. Interestingly, the VDE in *Chlamydomonas*, called the Chlorophycean VDE (CVDE), is found in the stroma, instead of the thylakoid lumen as is the case for the plant-type VDE, but still requires ∆pH for its activation (Li et al. 2016). Although the accumulation of zeaxanthin is a major contributor to the induction of both qE and qZ in plants, the zeaxanthin in *Chlamydomonas* has only a minor impact on the activation of qE (Niyogi et al. 1997b; Bonente et al. 2011). The accumulation of zeaxanthin still causes qZ in *Chlamydomonas*, but it is likely generated not only in LHCSR but also in other LHC proteins that bind zeaxanthin (Troiano et al. 2021).

For the qT component of NPQ, the reduced state of plastoquinone (PQ) in the thylakoid membrane, caused by an unbalanced level of excitation energy transferred to PSI and PSII, activates the STT7/STN7 kinase (Allen et al. 1981; Depège et al. 2003; Bellafiore et al. 2005), which phosphorylates LHCII and causes redistribution of excitation energy between PSI and PSII, allowing more energy to transfer to PSI and less energy to PSII (Rochaix 2011). The structural interaction of phosphorylated LHCIIs with PSI has been observed in cryo-electron microscopy analysis (Pan et al. 2018, 2021). Since HL also causes the accumulation of reduced PQ, the induction of both qE and qT processes likely happens at the same time. A certain level of synergistic processes between qE and qT has been previously observed in *Chlamydomonas*, as STT7 phosphorylates LHCSR3 in HL (Bonente et al. 2011; Allorent et al. 2013; Bergner et al. 2015; Scholz et al. 2019).

The molecular genetics and biochemistry of photoprotection mechanisms in *Chlamydomonas* have been extensively studied (Allorent and Petroutsos 2017; Rochaix and Bassi 2019). Yet, how thylakoid membrane macrostructures respond to HL has not been explored extensively. Previously, a transmission electron microscopy (TEM) study elucidated that the number of thylakoid stacks becomes lower in HL, and interestingly even lower when the cells were supplemented with carbon sources (acetate in media or 5% CO_2_ bubbling) (Polukhina et al. 2016). It is also suggested that the thylakoid structural changes in *Chlamydomonas* in HL are regulated by PSBS, whose function is still unclear (Redekop et al. 2020). In this study, we combined subdiffraction-resolution live-cell imaging and analytical membrane subfractionation to investigate the macroscale structural changes of thylakoid architecture during HL acclimation in *Chlamydomonas*. By using subdiffraction-resolution microscopy, we visualized a drastic change in the apparent thylakoid structure in the cells acclimated to HL. We also observed changes in the densities of isolated thylakoid membranes, which were analytically separated by density-dependent fractionation. We used *Chlamydomonas* mutants affecting qE and qT to observe how these two NPQ mechanisms are related to thylakoid structural changes in HL. The results indicated that neither qE nor qT components of NPQ are required for the observed macroscale structural changes of thylakoid membranes in HL conditions.

## Results

To observe thylakoid membranes in *Chlamydomonas* cells, we used a confocal microscope with Airyscan. This microscope has an array of 32 GaAsP photomultiplier tube detectors arranged in a hexagonal pattern and is capable of subdiffraction-resolution imaging by determining the microscope point spread function that is projected in the center of the array detectors (Huff et al. 2017). The spatial resolution achievable by Airyscan microscopy is theoretically lower than that of structured illumination microscopy (SIM). However, it is reported that Airyscan microscopy performs better than SIM for samples with a lower signal-to-noise ratio (Sivaguru et al. 2018). As compared to our previous study using SIM (Iwai et al. 2018), Airyscan images contained no obvious artifact due to lower signal-to-noise ratios. Therefore, these two microscopes are complementary techniques to obtain subdiffraction resolution.

**Fig. 1:**
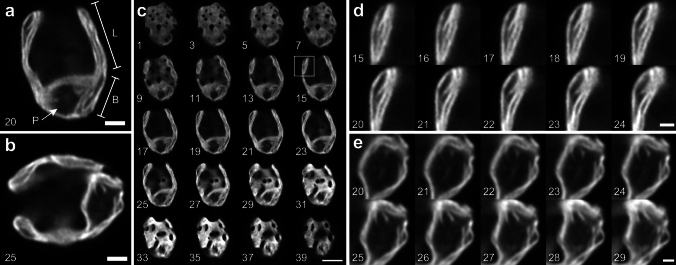
Airyscan images of *Chlamydomonas* acclimated to LL. **a**, **b** Representative images of the LL-acclimated cells observed by Chl fluorescence. L, lobe; B, base; and P, pyrenoid. **c** Optical serial sectioning of the cell (in **a**) through z-stack. **d** Enlarged images for optical sectioning of a lobe region (a square in **c**), showing layers of thylakoid membranes. **e** Enlarged images for optical sectioning of a pyrenoid region (in **b**), showing pyrenoid tubules. Numbers indicate slice numbers of z-stack images observed at every 100 nm. Scale bars = 2 µm (**a**, **b**), 5 µm (**c**), 1 µm (**d**, **e**)

**Fig. 2:**
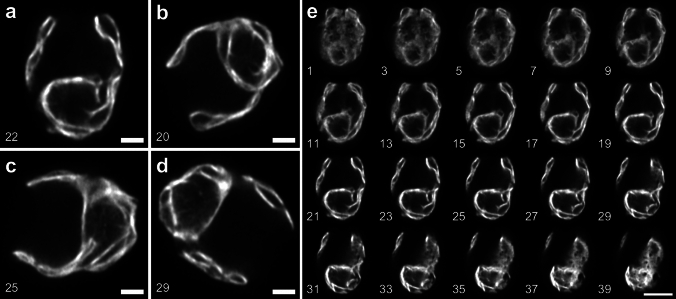
Airyscan images of *Chlamydomonas* acclimated to HL. **a**–**d**. Representative images of the HL-acclimated cells observed by Chl fluorescence. **e** Optical serial sectioning of the cell (in **a**) through z-stack. Numbers indicate slice numbers of z-stack images observed at every 100 nm. Scale bars = 2 µm (**a**–**d**), 5 µm (**e**)

We first observed live *Chlamydomonas* cells acclimated to LL (~30 µmol photons m^−2^ s^−1^) using the Airyscan microscope. The Airyscan images showed typical thylakoid structures in the cup-shaped chloroplast very similar to the ones previously observed using SIM (Iwai et al. 2018). The LL-acclimated cells typically showed undisturbed thylakoid layers with relatively little space between the layers throughout the lobe regions of the chloroplast (Fig. 1a, b). By optical sectioning through the z-axis, it was possible to observe the membrane surface patterns (Fig. 1c), which are usually difficult to obtain by TEM observation. The Airyscan imaging revealed smooth fluorescence patterns indicating continuous membrane regions with occasional empty spaces. Layers of thylakoid membranes in the lobe and thylakoid tubules were also visible (Fig. 1d, e). On the other hand, after HL (~350 µmol photons m^−2^ s^−1^) acclimation for 24 h, the overall thylakoid structures appeared to be thinner than that of the LL-acclimated cells. The thylakoid structures at the lobe showed fewer layers with more non-fluorescent spaces between the layers (Fig. 2a-d). Similarly, the thylakoid structures at the base became thin, and the space occupied by the pyrenoid became larger than that of the LL-acclimated cells. Optical sectioning showed that the surface pattern became rough and less dense due to the thinning of membranes (Fig. 2e). Further, the lobe regions appeared to be shorter and sometimes partially disappeared.

**Fig. 3:**
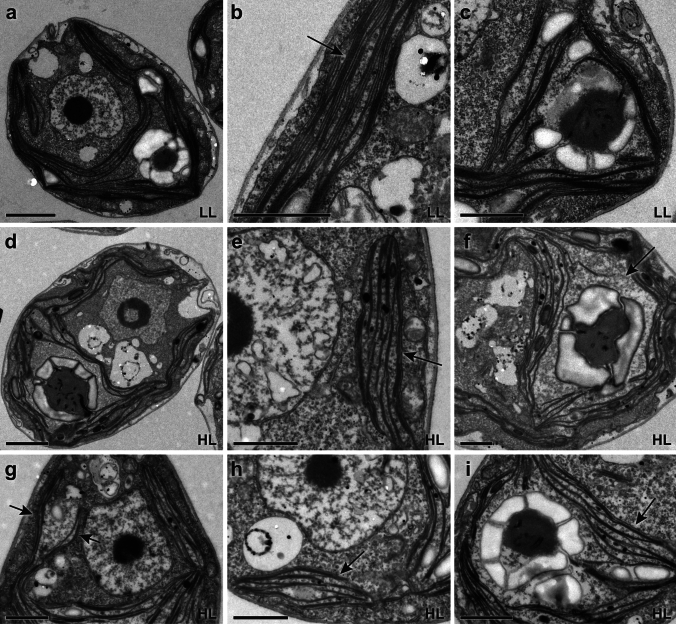
TEM images of *Chlamydomonas* acclimated to LL and HL. **a**–**c** Representative images of the LL-acclimated cells, showing an overall cell structure (**a**), thylakoid membranes (indicated by an arrow) at a lobe region (**b**), and a base region (**c**). Representative images of the HL-acclimated cells, showing an overall cell structure (**d**), thylakoid membranes (indicated by an arrow) at lobe regions (**e**, **g**, **h**), and enlarged stromal spaces (an arrow in **f**) around the pyrenoid at base regions (**f**, **i**). Scale bars = 2 µm (**a**, **d**), 1 µm (**b**, **c**, **e**–**i**)

We observed similar characteristics by using TEM. The TEM images of the LL-acclimated cells revealed that thylakoid membranes were continuously undisturbed with narrow spaces between the membranes (Fig. 3a). The membranes at the lobe regions were close to each other, and the ones at the base were also tightly associated near the pyrenoid (Fig. 3b, c). The TEM images of the HL-acclimated cells clearly showed more stromal spaces between the membranes throughout the thylakoid structures (Fig. 3d). In the lobe regions, there were larger spaces between the membranes, but also some membranes appeared to be associated tightly with each other (Fig. 3e, g, h). Interestingly, the enlarged space for the pyrenoid is actually the stromal space around the pyrenoid, as the size of the pyrenoid did not significantly change under HL (Fig. 3f). The membranes in the base regions also appeared more spaced out (Fig. 3f, i). These TEM results demonstrated consistency with the Airyscan images of the thinner thylakoid structures and more space between membranes observed in the HL-acclimated cells. It should be noted that the imaging observation shown in Figs. 1, 2, and 3 (and also the following results) were done using the *Chlamydomonas* cells grown in photoheterotrophic growth conditions (i.e., TAP media). A similar structural phenotype was observed when we used photoautotrophic growth conditions (i.e., HS media) as we have shown previously (Iwai et al. 2018).

**Fig. 4:**
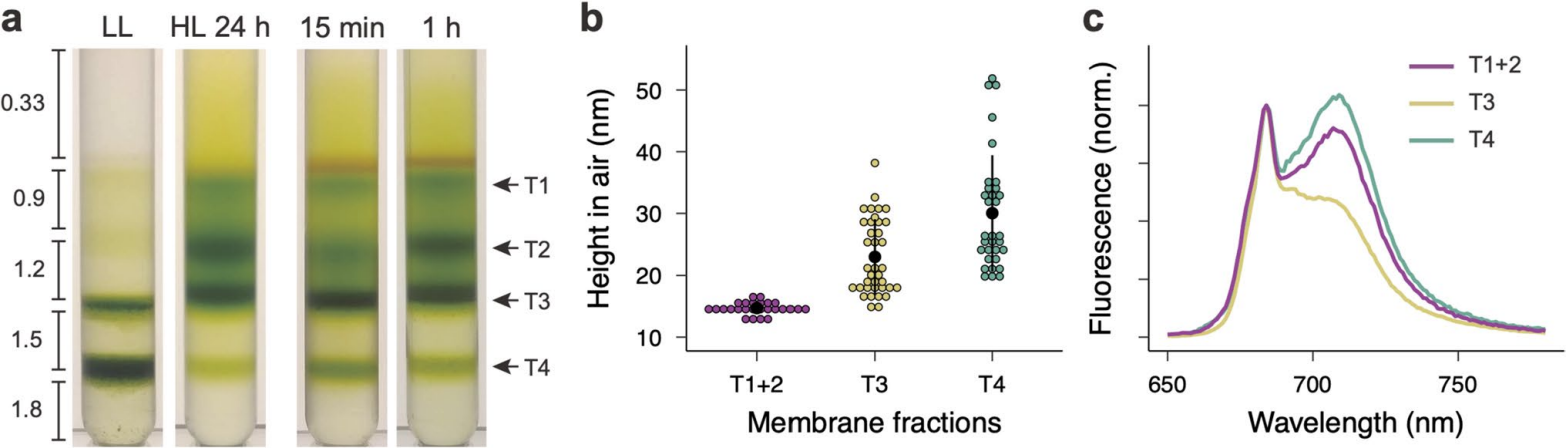
Analytical sucrose density-dependent membrane fractionation. **a** The cells acclimated to LL and HL were gently disrupted (a total of 0.5 mg Chl) and separated by sucrose step-gradient centrifugation. The results for the cells treated under HL for 15 min and 1 h were also shown. Numbers indicate the molar concentration of sucrose (see Methods for details). Different membrane fractions were indicated as T1-T4. **b** The height of isolated membranes in each fraction was measured by AFM. T1 + 2 and T3 fractions were from the cells acclimated to HL, and T4 fraction was from the cells acclimated to LL. **c** Chl fluorescence emission spectra at 77 K. Spectra were normalized at 684 nm. T1 and T2 (T1 + 2) and T3 were obtained from HL samples, and T4 was obtained from LL samples

To gain more insight into the thylakoid membrane structures, we performed analytical density-dependent fractionation of isolated thylakoid membranes using sucrose step-wise gradient centrifugation. This experiment showed that the chloroplasts in the LL-acclimated cells contained a thick green band of the densest membrane fraction (T4) and a less distinct green band of a lighter membrane fraction (T3) (Fig. 4a). In contrast, the chloroplasts in the HL-acclimated cells contained four green bands—the T4 fraction became least abundant, while the second lighter fraction (T2) and T3 fractions became abundant, and the lightest fraction (T1) became visible (Fig. 4a). An increase of carotenoids was also visible above the T1 fraction. The increase in the quantity of the lighter membrane fractions in the HL-acclimated cells is consistent with the imaging results that showed that overall thylakoid structures became thin in the HL-acclimated cells (Fig. 2). Surprisingly, an increase in T1-T3 fractions was already generated after 15 min of HL treatment (Fig. 4a). A gradual increase in T1 and T2 was observed from 15 min to 1 h of HL treatment, while the accumulation of T4 declined. The membrane heights measured using atomic force microscopy (AFM) also indicated that the T4 fraction contained thicker membranes than T3, and the membranes in T1 + T2 fractions were thinnest (Fig. 4b). The observed changes in height reflect the number of membrane stacks, as thylakoid membranes in *Chlamydomonas* also form the appressed and non-appressed membrane regions.

Recently, the lateral heterogeneity of the two photosystems has been shown in *Chlamydomonas* by cryo-electron tomography; PSII is located in the appressed regions, while PSI is physically segregated into the non-appressed regions (Wietrzynski et al. 2020). Chl fluorescence spectra at 77 K revealed that the T4 from LL contained the emission peaks for both PSII (~ 684 nm) and PSI (~ 710 nm), but the T3 from HL showed much less PSI emission than the T1 + T2 from HL (Fig. 4c). These results suggest that the T4 contains whole intact thylakoid membranes, T3 contains more appressed membranes, and T1 + T2 contains more non-appressed membranes and possibly fragmented membranes originating from both appressed and non-appressed membranes. Comparison of fluorescence intensities measured at 77 K between different membrane samples is rather complicated, because the spectra need to be normalized at a specific wavelength (i.e., 684 nm in our case). Also, when thylakoid membranes are isolated and/or disturbed, any resulting disconnection of antenna complexes will increase fluorescence emission, especially from LHCII as evidenced by the shoulder at around 676 nm in T1 + T2 (Fig. 4c). Therefore, it is likely that the T1 + T2 fraction contains disconnected LHCII, which could cause a higher fluorescence yield. This could explain why the relative PSI emission observed in T1 + T2 is lower than that of T4. Another explanation would be that T4 contains more intact membranes in which energy transfer to PSI (e.g., from LHCI to PSI, from LHCII to PSI, and/or spillover from PSII to PSI) occurs more efficiently than that of T1 + T2. Although our observation indicates that changes in membrane densities happened after only 15 min of HL treatment (Fig. 4a), HL acclimation could change the ratio between PSI and PSII, resulting in lower PSI emission observed in T1 + T2 relative to T4.

Because our membrane fractionation does not involve detergent solubilization (e.g., digitonin), and also *Chlamydomonas* thylakoids lack the distinct granum-like structures typically observed in land plant chloroplasts, we presume that our membrane fractions will not have a clear distinction of PSI and PSII populations. Despite that, our results from analytical density-dependent fractionation, the membrane heights, and Chl fluorescence emission at 77 K suggest that the HL-induced thylakoid structural changes involve membrane unstacking.

**Fig. 5:**
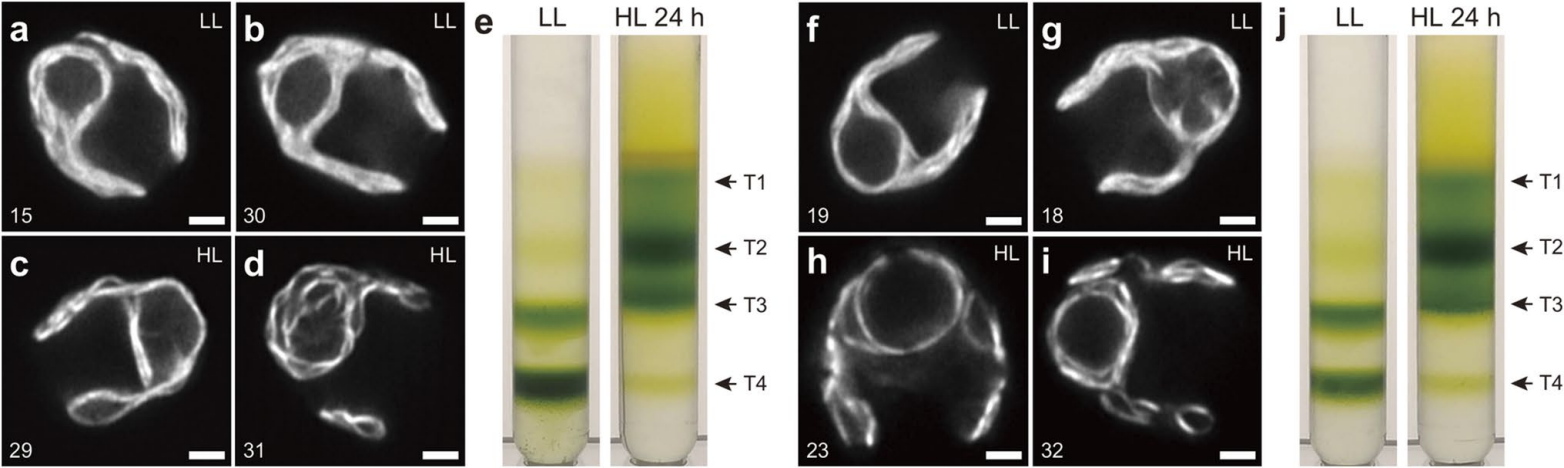
Thylakoid structural changes after HL acclimation in *npq4 lhcsr1* and *spa1-1*. Representative Airyscan images of the *npq4 lhcsr1* mutant cells acclimated to LL (**a**, **b**) and HL (**c**, **d**). **e** Analytical sucrose density-dependent membrane fractionation using the *npq4 lhcsr1* mutant cells acclimated to LL and HL were gently disrupted (a total of 0.5 mg Chl) and separated by sucrose step-gradient centrifugation (see Methods for details). Representative Airyscan images of the *spa1-1* mutant cells acclimated to LL (**f**, **g**) and HL (**h**, **i**). **j** Analytical sucrose density-dependent membrane fractionation using the *spa1-1* mutant cells acclimated to LL and HL were gently disrupted (a total of 0.5 mg Chl) and separated by sucrose step-gradient centrifugation (see Methods for details). Numbers (in **a**–**d**, **f**–**i**) indicate slice numbers of z-stack images observed at every 100 nm. Scale bars = 2 µm. Different membrane fractions were indicated as T1–T4

HL induces both ∆pH and *LHCSR* expression, which are essential components for NPQ induction in *Chlamydomonas*. Given its central role in NPQ and HL acclimation, a possible role of LHCSRs could be the induction of thylakoid structural changes during HL acclimation. Although the TAP-grown *Chlamydomonas* cells are considered to have a limited amount of LHCSR proteins, we tested the possibility directly by using two mutant lines that affect the accumulation of LHCSR proteins, *npq4 lhcsr1* and *spa1-1*. The *npq4 lhcsr1* line lacks both LHCSR3 and LHCSR1 proteins and is deficient in NPQ even after HL acclimation (Ballottari et al. 2016). The *spa1-1* line accumulates LHCSR1 at high levels even under LL (Gabilly et al. 2019). Airyscan imaging analysis revealed that the structural changes of thylakoid membranes in the *npq4 lhcsr1* line were very similar to that of WT after HL acclimation—thinned lobe structures, more space between membranes, and a larger stromal space around the pyrenoid (Fig. 5a–d). Analytical density-dependent fractionation of isolated thylakoid membranes also showed similar results—an increase in T1, T2, and T3 fractions and a decrease in T4 after HL acclimation (Fig. 5e). In the case of the *spa1-1* line, the stromal space around the pyrenoid was already enlarged before HL acclimation (Fig. 5f, g), which is due to high starch accumulation in the pyrenoid, which was not observed in WT (Figure S1). After HL acclimation, similar structural changes were observed in the *spa1-1* line relative to WT (Fig. 5h, i). Analytical density-dependent fractionation analysis also showed results similar to WT—more T4 fraction in LL and an increase in T1, T2, and T3 fractions after HL acclimation (Fig. 5j). Although differences in lateral heterogeneity of the two photosystems might exist between WT and each mutant, these results indicate that the thylakoid membrane structural rearrangement associated with HL acclimation occurs regardless of LHCSR accumulation. This is also consistent with a previous study showing that thylakoid unstacking occurs in the cells grown in photoheterotrophic growth conditions (supplemented with acetate or 5% CO_2_ bubbling), which causes a decrease in LHCSR protein accumulation (Polukhina et al. 2016).

**Fig. 6:**
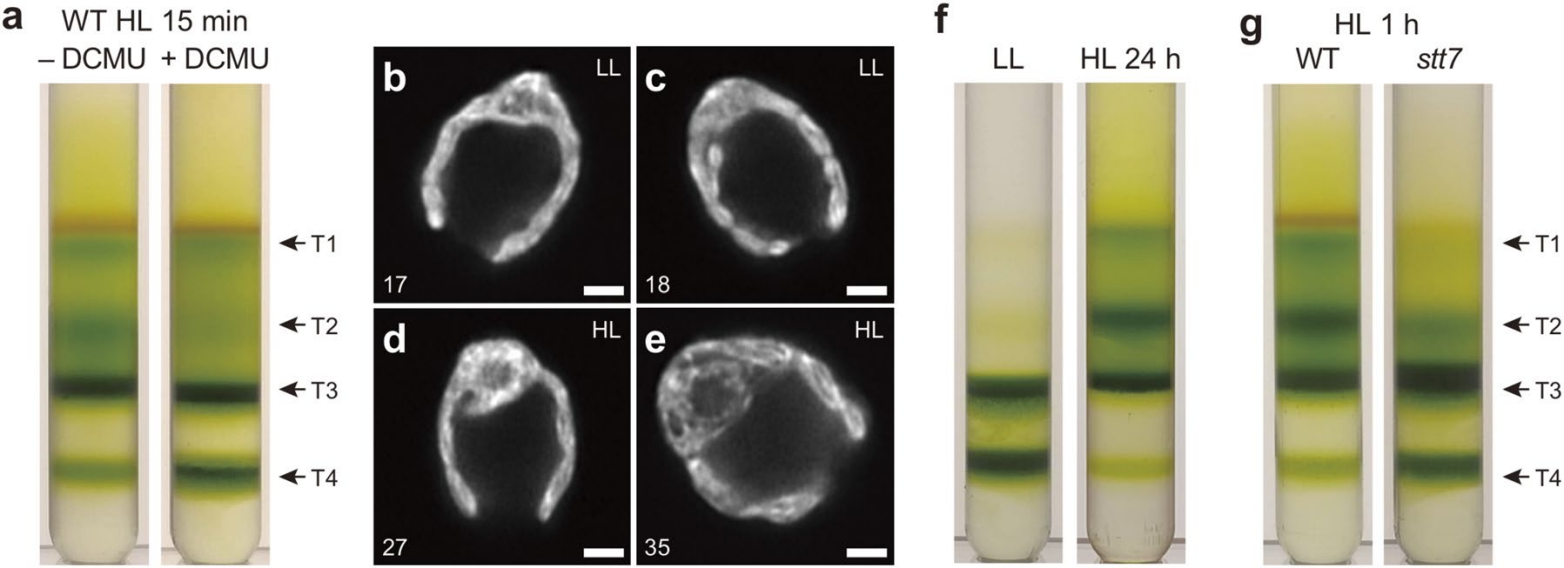
The effect of photosynthetic electron transfer and state transitions. **a** Analytical sucrose density-dependent membrane fractionation using WT cells treated with DCMU during 15 min of HL treatment were gently disrupted (a total of 0.5 mg Chl) and separated by sucrose step-gradient centrifugation (see Methods for details). Different membrane fractions were indicated as T1-T4. **b** Representative Airyscan images of the *stt7-9* mutant cells acclimated to LL (**b**, **c**) and HL (**d**, **e**) for 24 h. Numbers indicate slice numbers of z-stack images observed at every 100 nm. Scale bars = 2 µm. **f** Analytical sucrose density-dependent membrane fractionation using the *stt7-9* mutant cells acclimated to LL and HL (for 24 h) were gently disrupted (a total of 0.5 mg Chl) and separated by sucrose step-gradient centrifugation. **g** Analytical sucrose density-dependent membrane fractionation using the WT and *stt7-9* mutant cells acclimated to HL for 1 h were gently disrupted (a total of 0.5 mg Chl) and separated by sucrose step-gradient centrifugation

We considered the possibility that thylakoid membrane structural rearrangements in HL are related to photosynthetic electron transport itself. To investigate whether the HL-induced thylakoid structural changes are dependent on photosynthetic electron transport, we treated *Chlamydomonas* cells with 3-(3,4-dichlorophenyl)-1,1-dimethylurea (DCMU), which blocks electron transfer from PSII to PQ, inhibiting photosynthetic linear electron transport. Interestingly, the results demonstrated that the accumulation of T1 and T2 fractions was suppressed by DCMU treatment, and the accumulation of the T4 fraction increased relative to samples without DCMU (Fig. 6a). These samples were harvested after 15 min of HL to limit the severe photodamage that HL induces in DCMU-treated cells. The PQ redox state is known to affect state transitions (qT) in *Chlamydomonas*, which maintains the energy balance between PSI and PSII by dynamically redistributing excitation energy absorption between the two photosystems via reorganization of LHCII. DCMU treatment locks cells in the so-called “state 1 conditions” in which LHCII remains unphosphorylated (Allen et al. 1981), and excitation energy is preferentially transferred to PSII (Finazzi et al. 2002; Iwai et al. 2008).

To test whether the observations using the DCMU treatment were indeed related to state transitions, we used the *stt7-9* mutant, which has about sixfold lower abundance of the STT7 kinase than WT and does not perform state transitions (Depège et al. 2003; Cardol et al. 2009; Bergner et al. 2015). Intriguingly, there were less pronounced structural changes between LL- and HL-acclimated *stt7-9* cells than that observed in WT after 24 h of HL (Fig. 6b–e). However, analytical density-dependent fractionation analysis showed similar results to WT, indicating that thylakoid unstacking still occurred in *stt7-9* after 24 h of HL (Fig. 6f). Because the state transition is induced on the time scale of minutes, HL treatment for 24 h could have induced other long-term acclimation mechanisms possibly involved with qZ and/or qI. To observe the short-term effect on thylakoid structural changes, we treated both WT and the *stt7-9* mutant under HL for 1 h and analyzed them by density-dependent fractionation analysis. Interestingly, the result showed that the *stt7-9* cells had much less T1 and T2 and more T3 and T4 than in the WT (Fig. 6g). Thus, at least for a short-term response to HL, LHCII phosphorylation is necessary for the membrane unstacking that occurs in WT. This result is in agreement with the observation in *Arabidopsis*, in which LHCII phosphorylation causes dynamic thylakoid membrane unstacking (Fristedt et al. 2009; Wood et al. 2019).

## Discussion

Our observations demonstrated that macroscale structural changes of thylakoid membranes occur in *Chlamydomonas* during HL acclimation (Figs. 1, 2, 3, and 4). It also appears that Airyscan imaging and analytical density-dependent fractionation actually characterize different aspects of thylakoid structure in *Chlamydomonas*—the former visualizes overall membrane structural shapes but does not detect changes in thylakoid stacking, whereas the latter provides information specifically about the level of membrane stacking. According to the results using *npq4 lhcsr1* and *spa1-1*, the HL-induced structural changes are not directly correlated with the accumulation of LHCSR proteins in thylakoid membranes (Fig. 5). These results also reconfirmed that high qE capacity in *Chlamydomonas* is strongly correlated with the LHCSR protein level in thylakoid membranes but not with the macroscale structural changes of thylakoid membranes. Interestingly, the DCMU-treated cells did not show structural changes under HL, at least during a short period of time (15 min) of HL exposure (Fig. 6a). The DCMU treatment oxidizes the PQ pool by inhibiting the electron transport from PSII, which causes LHCII to remain unphosphorylated. We also observed that the lack of LHCII phosphorylation in the *stt7-9* mutant prevents membrane unstacking during a short-term (1-h) HL treatment (Fig. 6g). Thus, it seems likely that thylakoid membrane protein reorganization and thylakoid unstacking in short-term HL acclimation depend on LHCII phosphorylation. This is also consistent with a previous study showing that both ∆pH and LHCII phosphorylation are required to adjust the membrane height in *Arabidopsis* (Clausen et al. 2014). However, prolonged HL conditions (e.g., 24 h) eventually generated a similar level of unstacked membranes in the *stt7-9* mutant compared to WT (Fig. 6f). This could be because the *stt7-9* mutant has a leaky phenotype with roughly sixfold less STT7 kinase activity than WT (Bergner et al. 2015). Unexpectedly, Airyscan images did not show pronounced changes in thylakoid architecture in the *stt7-9* mutant after 24 h of HL treatment (Fig. 6d, e), suggesting that the observed changes in the macroscale membrane architecture and the level of membrane stacking occur independently.

It is worth mentioning the limitations of imaging analysis by observing Chl fluorescence. It is reasonable to assume that the structures observed by Chl fluorescence reflect thylakoid membranes, because these membranes are where Chl pigments are located. However, Chl fluorescence emission from PSI is difficult to observe at room temperature, especially with confocal microscopes, due to its higher excitation trapping than PSII (Trissl and Wilhelm 1993). Also, fluorescence intensity frequently fluctuates due to several factors, including NPQ and photosynthetic electron transport that can influence the interpretation of observed structural differences in thylakoid membranes due to different intensities of Chl fluorescence between LL- and HL-acclimated cells. Because of that, we did not perform statistical analysis on Chl fluorescence images, which could be unreliable for determining the thickness of thylakoid membranes. Therefore, it is necessary to use TEM to confirm the structural changes in thylakoid membranes observed by Airyscan microscopy (Fig. 3). Because our TEM images reveal similar structural differences in thylakoid membranes, we conclude that Airyscan microscopy is capable of visualizing the overall thylakoid structures sufficiently well to reveal the differences between the LL- and HL-acclimated cells. It should be noted that, although large stromal spaces between membranes are more visible in the HL-acclimated cells than in the LL-acclimated cells, it also appears that a lot of membranes in the HL-acclimated cells are associated closely with each other (Fig. 3e, g, h). This might suggest a possible alteration of refractive indices of the membrane, which would affect how much light is absorbed by LHC proteins (Capretti et al. 2019). However, because of the chemical fixation and negative staining done in conventional TEM, it is difficult to determine whether changes in such membrane compartmental spaces are correlated with native conditions. Although live-cell imaging techniques provide images at a lower resolution than EM techniques, an advantage is that it is feasible to evaluate a large number of samples, which is inherently more difficult to do with EM. In the future, it will be interesting to compare our live-cell, macroscale observations of thylakoid structure with advanced cryo-EM data that reveal microscopic details of the thylakoid compartment, membrane stacking, and protein organization in HL.

## Experimental procedures

### Strains, growth conditions, and HL treatment

*Chlamydomonas* WT strain 4A + (mt + , 137c background; CC-4051), *npq4 lhcsr1*, and *spa1-1*, and *stt7-9* mutants were grown in Tris–acetate-phosphate liquid media (Harris et al. 1989) as described previously (Niyogi et al. 1997a). Briefly, liquid TAP medium was placed in a sterile 250-mL polypropylene beaker covered with a polystyrene Petri dish bottom and shaken at 120 rpm, which allows light to be evenly distributed to the 50-mL *Chlamydomonas* culture as well as good aeration. The culture was adjusted to ~ 1 µg Chl/mL and incubated on a shaker under a constant light intensity at ~30 µmol photons m^−2^ s^−1^ (LL) at 25 °C for 3 d. For HL treatment, the LL-acclimated cells were diluted to 1 µg Chl/mL (for 24 h treatment) or 2 µg Chl/mL (for 15 min and 1 h treatments) in fresh TAP liquid media and incubated under a constant light intensity at ~350 µmol photons m^−2^ s^−1^ at 25 °C for the specified duration of time. For the 24-h incubation under HL, the culture was diluted again after 6 h of HL incubation to maintain a low cell density for sufficient HL treatments. To obtain enough cell density for sucrose density-dependent membrane fractionation, we used 40 beakers of 50-mL culture (total volume of 2 L). It should be noted that we tried using the *Chlamydomonas* cells photoautotrophically grown in HS minimal medium (Harris et al. 1989) in the same way, but the growth was too slow for us to have enough cell density in a 24-h HL treatment to perform density-dependent fractionation. Chl concentration was measured as described previously (Porra et al. 1989).

### Airyscan microscopy

We prepared *Chlamydomonas* cells for Airyscan microscopy according to the method previously used for SIM (Iwai et al. 2018). Briefly, the cultures grown in TAP liquid medium were centrifuged at 3000×*g* and 23 °C for 1 min. The pelleted cells were resuspended with 0.5% low-melting-point agarose in TAP medium and mounted between two coverslips placed in an Attofluor cell chamber. *Chlamydomonas* cells were observed using a Zeiss LSM 880 microscope equipped with the Airyscan detector with a Zeiss Plan-Apochromat 63 × /1.4 NA DIC M27 Oil objective. Chls were excited with 633 nm laser, and fluorescence was acquired through a 645 nm longpass filter. Image acquisition and analysis were done under the full control of ZEN software (Zeiss) and ImageJ software (US National Institutes of Health, https://www.nih.gov/).

### TEM

*Chlamydomonas* cells acclimated under LL and HL 24 h were centrifuged, resuspended, and fixed with 2% glutaraldehyde in TAP liquid media for 24 h at 4 °C in the dark. The fixed cells were washed and post-fixed with a sodium cacodylate buffer containing 1% (w/v) osmium tetroxide and 0.8% (w/v) potassium ferricyanide for 2 h. The fixed cells were rinsed with cacodylate buffer for 10 min twice. The fixed cells were dehydrated with increasing concentrations of acetone (35–100%). The dehydrated cells were then infiltrated and embedded in resin. Sections were cut to approximately 500 μm in diameter and 60 nm in thickness. The thin sections were collected on Maxtaform copper slot grids (2 × 1-mm oval hole) that had been coated with 0.5% formvar. Sections were dried and post-stained with 2% uranyl acetate for 7 min, followed by lead citrate for 7 min. The sections were dried and examined using a JEOL 1200 EX transmission electron microscope at 100 kV.

### AFM

Height of isolated thylakoid membrane was measured as described previously (Iwai et al. 2013). Briefly, a freshly cleaved mica surface was treated with adsorption buffer (10 mM Tris-HCl (pH 7.3), 150 mM KCl, 20 mM MgCl_2_). Then, isolated thylakoid membranes (0.25 µg Chl/µL) were added to the adsorption buffer on the mica surface and incubated for 10 min. After gentle wash with MilliQ water, the membranes were observed by using a commercial MFP-3D stand-alone AFM in tapping mode in air (Asylum Research). Silicon cantilevers with a length of 240 µm (*k* = 2 N/m; OMCL-AC240TS-C2, Olympus) were used. Averaged height was calculated from the membrane areas of at least 50 µm^2^.

### Analytical sucrose density-dependent membrane fractionation

*Chlamydomonas* cells were collected at 1870×*g* and 4 °C for 5 min (JLA9.1000, Beckman Coulter). The cells were resuspended at 0.125 mg Chl/mL with disruption buffer containing 25 mM MES-NaOH (pH 6.5), 0.33 M sucrose, 1.5 mM NaCl, 0.2 mM benzamidine, and 1 mM ε-aminocaproic acid at 4 °C. The cells were disrupted at 5 kpsi once using the MC Cell Disruptor (Constant Systems Ltd, Northants, UK). The disrupted cells (0.5 mg Chl in 4 mL) were loaded onto sucrose step-wise gradient, containing 0.9, 1.2, 1.5, and 1.8 M sucrose with 25 mM MES-NaOH (pH 6.5) and 1.5 mM NaCl (2 mL/each sucrose layer). The thylakoid membranes with different densities were analytically separated at 125,000×*g* and 4 °C for 1 h (SW 41 Ti, Beckman Coulter).

### Fluorescence emission spectroscopy at 77 K

Chl concentration of the sample obtained by analytical sucrose density-dependent membrane fractionation was adjusted to 5 µg/mL and placed in a glass tube and frozen in liquid nitrogen. Fluorescence emission was recorded at 77 K using FluoroMax-4 spectrophotometer (Horiba Scientific). Excitation wavelength was 440 nm with a 2-nm slit size. Emission wavelength measured was from 650 to 800 nm with a 2-nm slit size. Fluorescence emission for each sample was recorded consecutively three times to obtain averaged spectra.

## Supplementary Information

Below is the link to the electronic supplementary material.

Supplementary file1 (PDF 4484 kb)

## Data Availability

The data presented in this study are available from the corresponding author upon request.
