# Supplementary material for: Macroscale structural changes of thylakoid architecture during high light acclimation in Chlamydomonas reinhardtii
Source: Photosynth Res. 2024 Jan 5;162(2-3):427–37. doi: 10.1007/s11120-023-01067-1 (PMC11614958; doi:10.1007/s11120-023-01067-1)
Supplement: Supplementary file 1 — Supplementary file1 (PDF 4484 kb) [file 11120_2023_1067_MOESM1_ESM.pdf]

Supplementary material

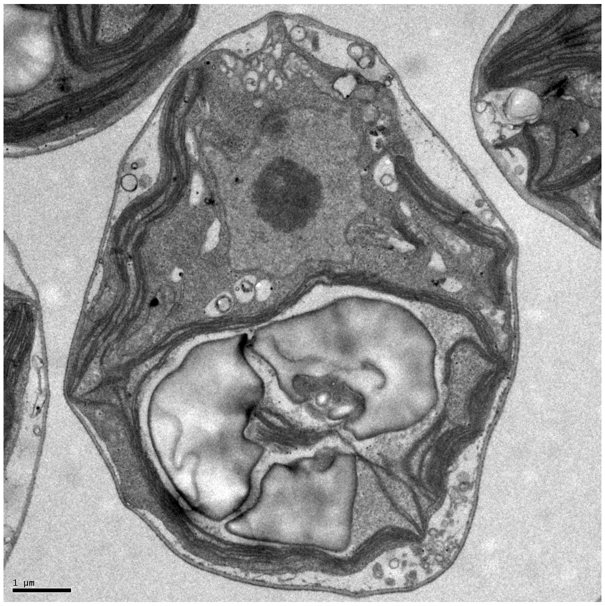

Figure S1. Representative TEM image of the *Chlamydomonas spa1-1* cell acclimated to LL conditions. Scale bar = 1 µm.
